# Novel methods of treating ovarian infertility in older and POF women, testicular infertility, and other human functional diseases

**DOI:** 10.1186/s12958-015-0001-8

**Published:** 2015-02-25

**Authors:** Antonin Bukovsky

**Affiliations:** The Institute of Biotechnology, Academy of Sciences of the Czech Republic, Prague, Czech Republic

## Abstract

*In vitro* maturation (IVM) and *in vitro* fertilization (IVF) technologies are facing with growing demands of older women to conceive. Although ovarian stem cells (OSCs) of older women are capable of producing *in vitro* fresh oocyte-like cells (OLCs), such cells cannot respond to IVM and IVF due to the lack of granulosa cells required for their maturation. Follicular renewal is also dependent on support of circulating blood mononuclear cells. They induce intermediary stages of meiosis (metaphase I chromosomal duplication and crossover, anaphase, telophase, and cytokinesis) in newly emerging ovarian germ cells, as for the first time demonstrated here, induce formation of granulosa cells, and stimulate follicular growth and development. A pretreatment of OSC culture with mononuclear cells collected from blood of a young healthy fertile woman may cause differentiation of bipotential OSCs into both developing germ and granulosa cells. A small blood volume replacement may enable treatment of ovarian infertility *in vivo*. The transferred mononuclear cells may temporarily rejuvenate virtually all tissues, including improvement of the function of endocrine tissues. Formation of new follicles and their development may be sufficient for IVM and IVF. The novel proposed *in vitro* approaches may be used as a second possibility. Infertility of human males affects almost a half of the infertility cases worldwide. Small blood volume replacement from young healthy fertile men may also be easy approach for the improvement of sperm quality in older or other affected men. In addition, body rejuvenation by small blood volume replacement from young healthy individuals of the same sex could represent a decline of *in vitro* methodology in favor of *in vivo* treatment for human functional diseases. Here we propose for the first time that blood mononuclear cells are essential for rejuvenation of those tissues, where immune system components participate in an appropriate division and differentiation of tissue stem cells. If needed, small blood volume replacement from distinct young healthy individuals could be utilized in six month intervals for repair of young altered or aged reproductive and other tissue functions. Systemic and local use of honey bee propolis tincture is an alternative option for functional rejuvenation of some tissues.

**Electronic supplementary material:**

The online version of this article (doi:10.1186/s12958-015-0001-8) contains supplementary material, which is available to authorized users.

## Editorial

The article series “IVF - the past, current development and its future” [1] deals with IVF advances in research over the past 10 years and its expected development (reviewed in a blog by Natasha Salaria [2]). Of particular interest are questions on how to improve IVF results in older women and solve infertility in women with premature ovarian failure (POF) or other types of ovarian infertility. In contrast to natural menopause, women diagnosed with POF may undergo unpredictable ovarian function leading to intermittent and unpredictable menses in 50% of cases, and conceive and deliver a child in ~5 to 10% of cases. In addition, other authors use the term primary ovarian insufficiency (POI) [3] instead of POF [4]. Most POF women, however, lack ovarian follicles and there are no practical evidences that their infertility can be solved by IVF, except for oocyte/embryo donation cycles [5,6].

At present, it is obvious that the IVF approach is subjected to the increased demands of older women. In developed world, “graying” of infertility populations and of infertility services was recently reported by Norbert Gleicher and colleagues as “an impeding revolution nobody is ready for” [7]. In this IVF series article authors reviewed this approach indicating that IVF live birth rates decrease to close to zero after 42 years, with no clinical pregnancies between 46–53 years.

Heide Schatten and colleagues [8] deal with a vital role of mitochondria in oocyte functions. Oocytes of women affected by metabolic disorders, such as diabetes or obesity and oocyte aging, can be improved by transfer of mitochondria from cells with mitochondrial integrity into mitochondria-impaired oocytes.

Deepa Bhartiya and colleagues [9] reviewed *in vitro* approaches for the development of oocytes and sperm from embryonic stem cells (ESC), induced pluripotent stem cells (iPS cells), ovarian stem cells (OSCs), pluripotent stem cells (PSCs), spermatogonial stem cells (SSCs), and very small embryonic-like stem cells (VSELs). They concluded that “Scientific community needs to slow down, re-think and make efforts to exploit clinical potential of pluripotent stem cells (VSELs) and progenitors (SSCs and OSCs) which exist in the adult gonads as an alternate option to ES/iPS cells.” They proposed: “Rather than the existing concept of *in vitro* differentiation of stem cells into oocytes and sperm for assisted reproduction, it would be ideal to manipulate VSELs that survive oncotherapy *in vivo* to achieve restoration of gonadal function (since they exist in menopausal/POF ovary and also in azoospermic human testis)” and “This approach will give rise to autologous gametes, with no associated ethical/regulatory constraints and epigenetic/genetic issues may not exist by avoiding *in vitro* culture”.

### Rationale for using *in vitro* developed oocyte-like cells and possible IVM/IVF developments for clinical use

First report of *in vitro* differentiation of oocyte-like cells (OLCs) from ovarian surface epithelium (OSE) cells of adult human ovaries was published ten years ago [10].

To better understand a possibility of clinical use of such *in vitro* developed OLCs for IVF purposes, or additional possible IVM/IVF developments, one will need to understand and review, including construction of this article, how the germ and granulosa cells are developed and processed in fetal mammalian ovaries and renewed in later adult life, as well as conditions leading to atresia of old primary follicles and formation of new primary follicles for cyclic follicular renewal during the prime reproductive period, but not thereafter.

In 1966 Erickson [11] reported that the numbers of primary follicles in bovine ovaries remain stable until 7 years of age, and decline significantly thereafter, when reproductive capacity begins to decline. Block [12] and Gougeon *et al.* [13] showed that in women between 18-38 ± 2.4 years of age, it is not possible to detect significant changes in the number of oocytes and follicles.

If we take into account that at least 60% of oocytes in adult human ovaries are in various stages of degeneration [14], we may conclude that without follicular renewal the ovarian function will cease within a few months. However, in aging ovaries, the elimination of degenerating ovarian structures appears to be altered along with a lack of regression of other temporary ovarian structures (e.g. corpora lutea [15]), possibly due to age-induced alterations of immune system functions, beginning from the age of 35 years [16]. Due to the absence of corpora lutea during fetal immune adaptation toward self tissues, they are considered to be a ‘graft’, which results in their cyclical rejection during menstrual cycles in adulthood [17]. The corpus luteum rescue during pregnancy accompanies immune tolerance of fetal allograft, and both effects required for successful pregnancy are considered to be caused by trophoblast-derived chorionic gonadotropin and other endocrine factors [18,19]. The cyclicity of ovarian function is considered to be primarily dependent on the induction of a specific cyclic immune response to the ovary [20]. In older females, however, the regression of corpora lutea is affected, and corpora lutea persist due to the developing senescence of the immune system [15]. Due to the developing immune senescence, atresia may not affect resting primary and growing preantral follicles in aging ovaries [13], and such follicles may persist in spite of accumulation of environmental and genetic alterations of oocytes in older women.

It is important to indicate that older fathers may contribute just as much as older mothers to the dramatic increase in Down syndrome risk in older couples. A recent study found that fathers older than 40 years of age were responsible for up to 50% of the rise in Down syndrome risk when the mother was also over 40 [21]. Of note, the number of births in couples over 35 years of age has more than doubled in the last 20 years and this has raised questions about the role of paternal age in the risk of genetic abnormalities and birth defects [21].

### Formal terms related to the ovarian cellular conditions and functions

In this article, the following formal terms are used: **Ovarian Stem Cells** (OSCs) for the bipotential stem cells causing granulosa and germ cells origin; ovarian **Fetal germ cells** and **Adult germ cells** - (see Ref. [22] for testicular fetal and adult germ cells) instead of non-existing persisting primordial germ cells, germline stem cells, or oogonial stem cells. **New primary follicles** (see Ref. [23]) instead of non-existing primordial ovarian follicles; **Resting primary follicles** instead of non-existing resting primordial follicles; **Growing preantral follicles**; **Small antral follicles** (suitable for IVM); and **Large antral follicles** (suitable for IVF).

Ovarian VSELs are, in reality, cytokeratin (CK) and major histocompatibility complex class I (MHC-I) positive OSE cells [24], later called ovarian stem cells [10], each measuring about 4 microns in diameter. They lose the CK and MHC-I expressions when transformed into fetal germ cells or adult germ cells, but not when transformed into granulosa cells [24,25].

Ovarian stem cells regenerate from their CK positive fibroblast type cell precursors present after the birth in the developed tunica albuginea (TA) (see Figure two in Ref. [23] and below). Genetically, CK and MHC-I positive OSCs are precursors of CK and MHC-I positive granulosa cells. The role of embryonic primordial germ cells is to commit OSCs for production of fetal and adult germ cells (see Figure five in Ref. [26]).

### Stem cell commitment by embryonic primordial germ cells

The commitment by primordial germ cells gives OSCs a possibility to differentiate into distinct cell types, including neural/neuronal cells [27]. Certain pluripotency of stem cells in some other tissues may also be established by primordial germ cell settlement during the embryonic period, since they circulate in embryonic blood vessels [28]. It has been reported that those OLCs expressing oocyte markers, such as Oct4, Vasa, Bmp15, and Scp3, may even be developed from porcine skin stem cells [29]. However, this line of research has been abandoned since OLCs remain unable to undergo maturation or fertilization because they do not complete meiosis [30].

### Roles of the immune system in ovarian function

Since the late 1970s, we have been involved in analyzing the role of the immune system in the regulation of normal ovarian function [20]. In 1995 we showed for the first time that during the reproductive life of women, OSE cells form *in vivo* new granulosa cell nests and, with the assistance of immune system cells, they are a source of new adult germ cells for follicular renewal [24]. Therefore, the OSE cells represent *in vivo* bipotential OSCs.

### Steps required for follicular renewal in adult human ovaries

To deal with the treatment of ovarian infertility, the researches and physicians should be aware of the following issues occurring in adult (and fetal) ovaries which are listed below. During follicular renewal in adult human ovaries, the following conditions were observed and interpreted: [10,17,23-27,31-35] (see also Figures below):

1. Availability of adult germ cells passing meiosis I metaphase with chromosomal crossover.
2. Availability of adult germ cells differentiated into the meiosis I telophase and cytokinesis stage.
3. Their migration guided by the CD14 and DR+ MDCs, and entering into blood venules in the upper ovarian cortex for vascular transport to the lower ovarian cortex (about 1000 microns distance).
4. Differentiation and association of granulosa cell nests with cortical venules in the lower ovarian cortex
5. Assembly of circulating oocytes with granulosa cell nests to form new primary follicles.
6. Formation of the oocyte’s primary and secondary Balbiani bodies (see below) from granulosa cells, which supply new specific organelles needed for early oocyte development and advanced oocyte growth with meiosis II resumption.

Absence of any of these steps, some of which require proper functioning of the ovary-committed immune system components and vegetative innervation, may result in functional infertility, which would be difficult, if not impossible, to treat by currently available IVM and IVF approaches.

### Origin and development of ovarian germ cells

**Figure 1:**
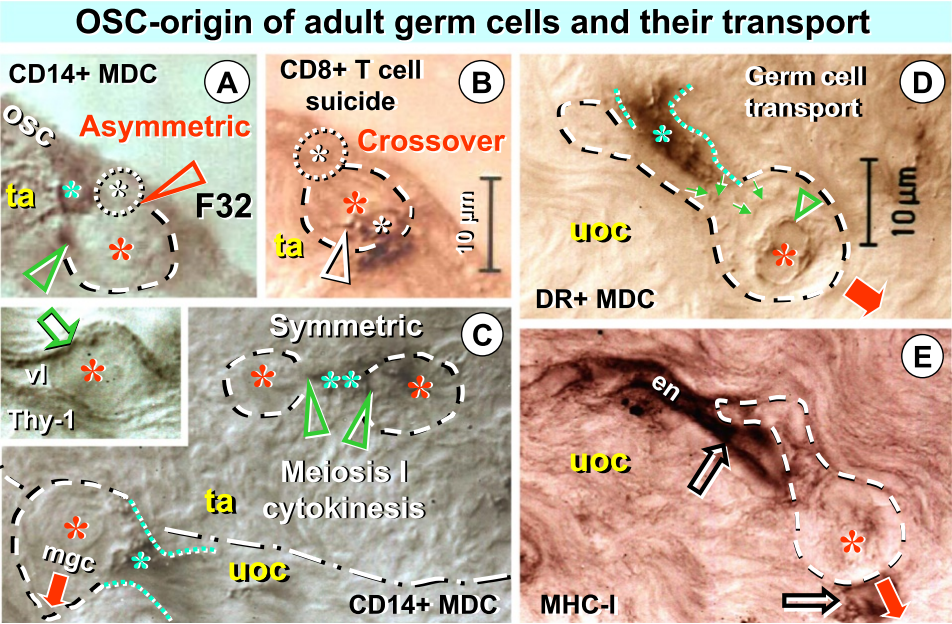
**Immune type cells influence commitment of OSCs in adult human ovary (age 32 years, midfollicular phase). A)** Primitive CD14 MDC (green asterisk) associates with a small OSC (yellow asterisk and dotted circle) accompanying origination (green arrowhead) of a larger germ cell (red asterisk and dashed line) by asymmetric division of OSC (red arrowhead). **B)** A serial section shows that asymmetric division is also accompanied by CD8 T cell (white asterisk) entering germ cell and exhibiting extensions (white arrowhead). **C)** Divided primitive CD14 MDC (green asterisks) accompany (green arrowheads) symmetric division (meiosis I cytokinesis) of germ cells (red asterisks) in the TA (ta) and germ cells moving (arrow) into the adjacent upper ovarian cortex (uoc). Inset shows a blood venule in the upper ovarian cortex with Thy-1 differentiation protein expression by vascular pericytes (arrow) and venule lumen (vl) containing a germ cell (red asterisk). **D)** Germ cell transport in the upper ovarian cortex is associated with an attached activated (DR+) MDC (green asterisk and dotted lines) releasing DR+ cytoplasmic particles (green arrows) which accumulate at the surface of the germ cell nucleus (arrowhead). **E)** Endothelial cells (en and open arrows) of a venule in the upper ovarian cortex exhibit MHC-I expression, which is not expressed by associated migrating (red arrow) germ cell (asterisk). See text for additional details. Adapted from Ref. [24], with a permission: © Blackwell Publishing, Oxford, UK.

Figure 1 demonstrates that adult germ cells do not originate from persisting oogonia (so called primordial germ cells or oogonial stem cells) but from cytokeratin positive OSCs. Origin of adult germ cell is initiated by an association of a primitive CD14+ monocyte derived cell (MDC) causing OSC division, and by CD8+ T cell causing that one of resulting daughters begins to develop in a distinct way, via asymmetric division [36]. The CD8 T cell is also expressing HLA DR (see below) causing its activation. It invades the developing adult germ cell and causes expression of PS1 meiotically expressed carbohydrate protein [37] accompanying chromosomal duplication and crossover during meiosis I prophase and metaphase (see below).

The CD14+ MDC binds to developed adult germ cell, causes meiosis I cytokinesis, and divides in the TA. Next, the primitive CD14 cells become activated (HLA DR+) and transport adult germ cells from the TA to blood venules in the upper ovarian cortex. In other words, those MDCs coming to the ovary from TA venules participate on asymmetric origin of germ cells from OSCs, cause symmetric meiosis I division of germ cells, and forward the germ cells toward the cortical venules, back from where the MDCs entered the ovarian upper cortex and the TA.

**Figure 2:**
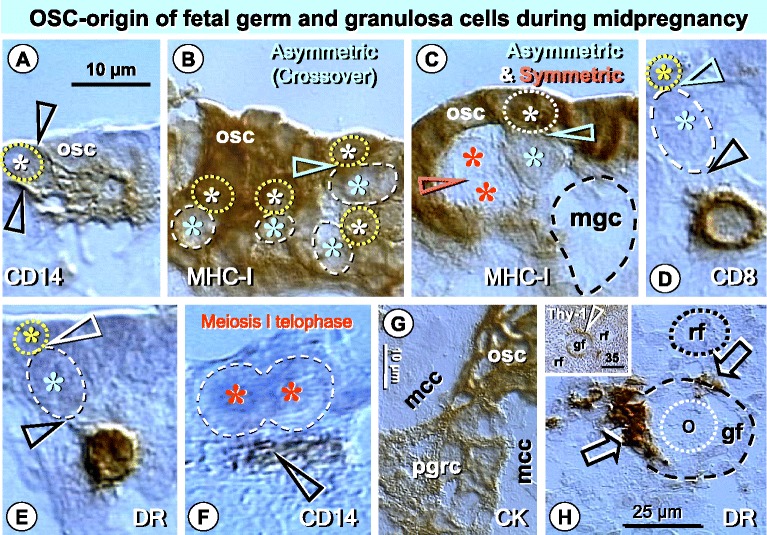
**Formation of fetal germ cells, granulosa cells, and follicular development in midpregnancy human fetal ovary. A)** CD14 MDC interacts (arrowheads) with an OSC (yellow asterisk and dotted circle) prior to the asymmetric division. **B)** Numerous MHC-I depleted fetal germ cells (blue asterisks and dashed circles) originating by asymmetric divisions (arrowhead) from MHC-I+ OSCs (yellow asterisks and dotted circles). **C)** Asymmetric division (blue arrowhead) is followed by a symmetric division of MHC I depleted germ cells (red asterisks and arrowhead). This is followed by the development of ameboid shape fetal moving germ cell (mgc - dashed line, no hematoxylin counterstain) entering adjacent ovarian cortex. Asymmetric division is accompanied by CD8 **(D)** and DR+ **(E)** T cell. **F)** CD14 MDC (arrowhead) accompanies symmetric division of germ cell during meiosis I telophase. **G)** Development of primitive granulosa cells (pgrc, note lower CK expression) from ovarian stem cells between mesenchymal cell cords (mcc). **H)** DR+ MDC accompany (arrows) fetal growing follicle (gf) but not the resting follicle (rf). Inset shows association of Thy-1+ pericytes (arrowhead) with a growing but not resting follicles. Bar in A for A-F. Adapted from Ref. [25] with a permission: © Springer US.

Figure 2 shows that primordial germ cells do not persist even in fetal ovaries, but new germ cells originate from CK+ OSCs with the assistance of immune cells. Multi-layered fetal OSC epithelium exhibits an emergence of numerous fetal germ cells with a depletion of MHC-I expression. After asymmetric division with crossover of chromosomes, a symmetric meiosis I division of germ cells soon follows and mowing germ cells leave the OSC epithelium toward granulosa cells in the adjacent ovarian cortex to form fetal primary follicles (see Ref. [25,34] for details). Note the absence of the TA, containing OSC and granulosa cell precursors, which is formed in perineonatal ovaries around the birth (review and data are available in Ref. [23]), see also below.

Like in adult ovaries, the emergence of germ cells by asymmetric division is accompanied by CD8 T cells expressing HLA DR. That was also observed in T cells undergoing suicide during differentiation of epithelial cells [36].

Beside origin of germ cells, OSCs also are a source of primitive granulosa cells that emerge between ovarian mesenchymal cell cords and contribute granulosa cells to the formation of fetal primary follicles (see Ref. [25,34] for details). Activated (DR+) MDCs accompany growing fetal follicles, but not resting ones. Thy-1+ vascular pericytes also associated with growing but not with resting follicles, like in adult ovaries [24].

**Figure 3:**
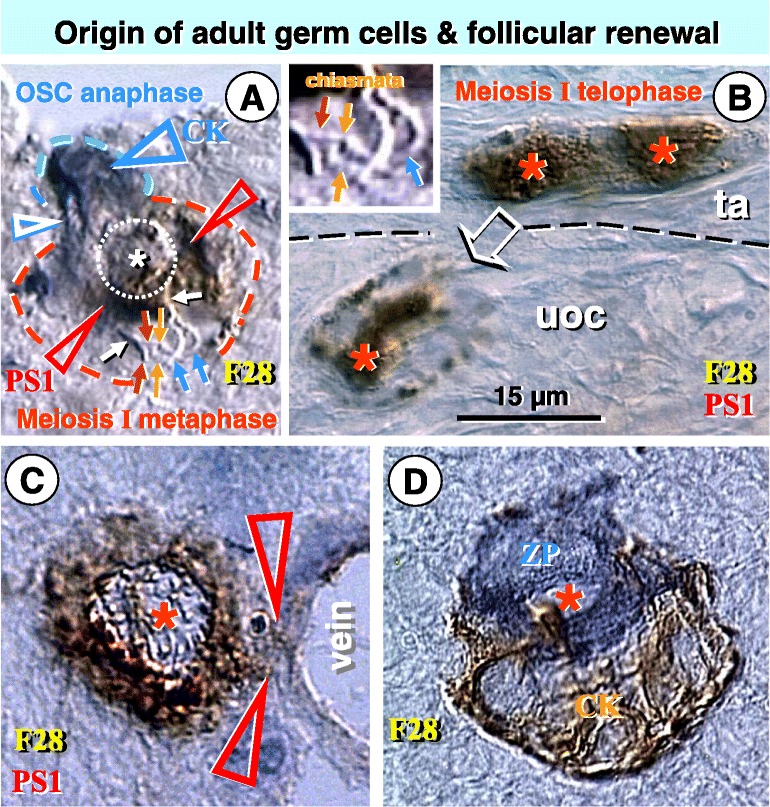
**Origin, meiosis I, and migration of human adult germ cells, and follicular renewal in a 28 year old women (F28). A)** Dual color IHC of asymmetrically dividing OSC with CK+ (blue dashed line) OSC daughter and PS1+ (red dashed line) germ cell daughter. CK+ OSC daughter chromosomes (white arrowhead) move to the OSC end during mitotic OSC anaphase. Germ cell chromosomes (white arrows) duplicate by DNA replication (red and blue arrows) and exhibit metaphase I sister chromatid crossover (orange arrows) during meiosis I metaphase. White asterisk and dotted circle indicate PS1+ putative CD8/DR+ suicidal T cell within the germ cell (see Figures 1B, 2D,E and Ref. [36]). **B)** In the TA (ta) the symmetrically dividing germ cell exhibits strong nuclear (asterisks) PS1 expression, which accompanies the meiosis I telophase. Arrow indicates a germ cell moving from the TA to the upper ovarian cortex (uoc). Inset shows a detail of chromosomal crossover (orange arrows) from panel A. Red and blue arrows indicate interacting sister chromatids. **C)** Germ cell with a diminution of nuclear and increase of cytoplasmic PS1 staining. It begins to enter (arrowheads) the vein in the upper ovarian cortex. **D)** Early stage of new primary follicle formation with ZP (blue color) expression of a small oocyte captured by the CK+ (brown color) granulosa cell nest. See details in the text. Adapted from Ref. [3]: © Antonin Bukovsky.

Figure 3 demonstrates that PS1 meiotically expressed carbohydrate protein [37] is expressed by ovarian adult germ cells and small oocytes, and association of zona pellucida (ZP) expressing oocyte with CK+ granulosa cell nest during follicular renewal in a 28 year old women. Dual color immunohistochemistry (IHC) shows asymmetric division of OSC during mitotic anaphase. The dividing cell consists of a small blue CK+ OSC and PS1+ brown large germ cell. The OSC chromatids are moving during mitotic anaphase to the OSC daughter end. A putative CD8/DR+ suicidal T cell (see Figures 1B, 2D,E, and Ref. [36]) is expressing PS1 and induces PS1 expression in its surroundings (red arrowheads, Figure 3A). In reality, the presence of CD8/DR+ T cell within emerging germ cell causes that the developing germ cell is already in the meiosis I metaphase, when the chromosomes duplicate and homologous chromosomes exchange genetic information (chromosomal crossover) before meiosis I anaphase. Inset in Figure 3B shows a detail of the chromosomal crossover - orange arrows indicate chiasmata of sister chromatids during meiosis I metaphase (see Ref. [38] for review).

Strong nuclear PS1 expression was detected in adult germ cells in meiosis I telephase indicating involvement of PS1meiotically expressed carbohydrate protein in meiosis I events. Germ cells migrating from the TA to the upper ovarian cortex exhibit a diminution of nuclear PS1 expression. Germ cells in the upper ovarian cortex resemble small oocytes and exhibit strong cytoplasmic PS1 expression only. They enter the adjacent cortical veins.

The vascular transport of small oocytes in adult human ovaries is usually required for later association with granulosa cell nests in the lower ovarian cortex (about 1000 microns from the ovarian surface). In contrast, human fetal ovaries are much smaller and do not require vascular transport, since oocytes attain developed granulosa cells by their own movement [25]. The same applies for adult small laboratory rodents, like rats [39]. Figure 3 also shows follicular renewal with blue zona pellucida staining of a small oocyte and brown staining of CK+ granulosa cell nest in the lower ovarian cortex of the same 28 year-old woman.

Some exemptions may, however, occur on adult human ovaries. When the OSC epithelial crypts reach by invagination the lower ovarian cortex, they produce germ cells able to move by their own movement (without vascular transport) to the neighboring granulosa cell nests to form new primary follicles. The granulosa nests reach the lower ovarian cortex within stromal sprouts migrating from the upper cortex under the influence of DR+ MDC (see Figure twelve in Ref. [23]).

**Figure 4:**
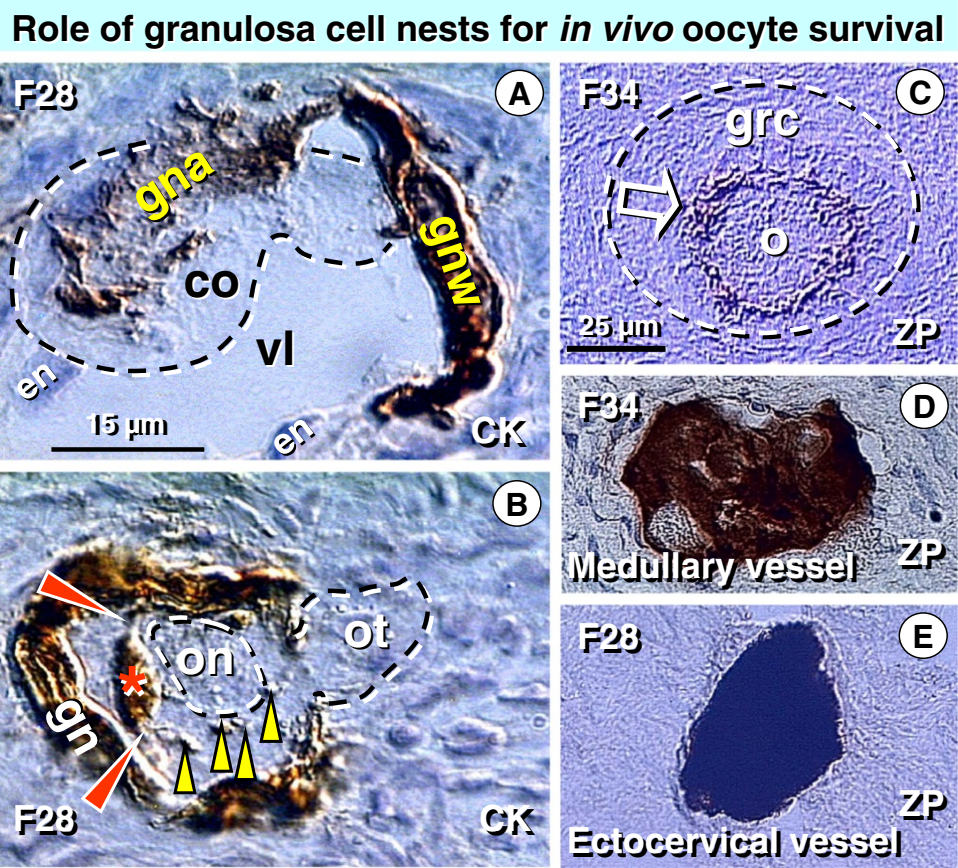
**Follicular renewal in adult human ovary and intravascular degeneration of germ cells unattended with granulosa cells. A)** Ovarian vein in the lower ovarian cortex lined by endothelial cells (en) and CK+ granulosa nest wall (gnw). In the vein lumen (vl) the granulosa nest wall extends a granulosa nest arm (gna) capturing the circulating oocyte (co). **B)** Granulosa nest (gn) during formation of the new primary follicle with captured oocyte. Granulosa cells penetrate the ooplasm (red arrowheads) during the primary Balbiani body (asterisk) formation adjacent to the oocyte nucleus (on). CK+ granulosa nest particles (yellow arrowheads) are already dispersed within the oocyte, which still exhibits oocyte tail (ot) outside of the new primary follicle. **C)** Growing follicle (dashed line circle), with granulosa cells (grc) and oocyte (o) with ZP expression at the oocyte surface (arrow). **D)** Degenerating oocyte in a medullary vein from the same ovary as in panel C exhibits a strong cytoplasmic ZP expression. **E)** Heavily ZP+ degenerating oocyte from 28 year-old fertile woman found in the extra ovarian (uterine ectocervix) vein of a patient with follicular renewal shown in panels 4A and 4B. Panels A and B adapted from Ref. [23] : © Antonin Bukovsky; panels C-E adapted from Ref. [41] , with a permission: © Elsevier/North-Holland Biomedical Press.

Figure 4 shows an oocyte-granulosa cell nest assembly with primary Balbiani body formation in the identical ovary of a 28 year old woman, and the fate of circulating oocytes lacking association with granulosa cells. Granulosa cell nests for follicular renewal are formed during follicular phase of ovarian cycle from the OSCs [24], with assistance of DR+ MDCs [23,24,26]. After the nests reach venules in the lower ovarian cortex, CK+ granulosa nest walls replace vein’s endothelial cells and the granulosa nest arm captures a circulating oocyte from the vascular lumen (Figure 4A).

Figure 4B demonstrates that the CK+ oocyte’s primary Balbiani body adjacent to the oocyte nucleus is already formed by granulosa cell nest extensions penetrating into the ooplasm very early during follicular renewal, at the time when the oocyte tail is still outside of the developing new primary follicle. Cytokeratin positive particles within the ooplasm suggest a fast release of the Balbiani body components. It contains additional organelles, such as Golgi vesicles, endoplasmic reticulum membranes, and nascent forms of smooth endoplasmic reticulum, needed by oocytes to develop further [40]. For additional examples of sequential stages of oocyte-nest assembly and primary Balbiani body formation see Figures five to eight in Ref. [23].

Before and after reaching the new primary follicle stage, oocytes show ZP expression at the oocyte surface [41]. Oocyte surface ZP expression in a growing preantral ovarian follicle of a 34-year old woman is presented in Figure 4C. In the same women, the medullary vessels show degenerating oocytes with heavy cytoplasmic ZP expression (Figure 4D).

During follicular renewal, ZP+ degenerating oocytes were even detected in the vessels outside of the ovary (Figure 4E) of the same 28 year old woman with follicular renewal shown here. This indicates that, during human follicular renewal, oocytes circulate through the blood stream, and those which are unable to associate with available ovarian granulosa cell nests degenerate. Thus during follicular renewal, the number of newly formed adult germ cells is significantly higher than the number of newly formed granulosa cell nests [23]. In other words, the number of developed granulosa cell nests in a given ovarian cycle determines how many new follicles will be formed. Consequently, if no granulosa cell nest are formed, no follicular renewal occurs (e.g., after 38 ± 2 years of age). Even if adult germ cells are still available in aging mammals [42], the lack of uncommitted granulosa cell nests causes a lack of formation of new primary follicles.

Functional tissue longevity depends on the timing of their appearance during early periods of life - the tissues appearing very early during immune adaptation, like the hart, can in humans function for one hundred years [43]. The presence of germ cells in aging mammals is caused by their early appearance - during embryonic adaptive period of development, while lack of granulosa cells in aging mammals is caused by their later appearance - during the fetal adaptive period of development [34].

**Figure 5:**
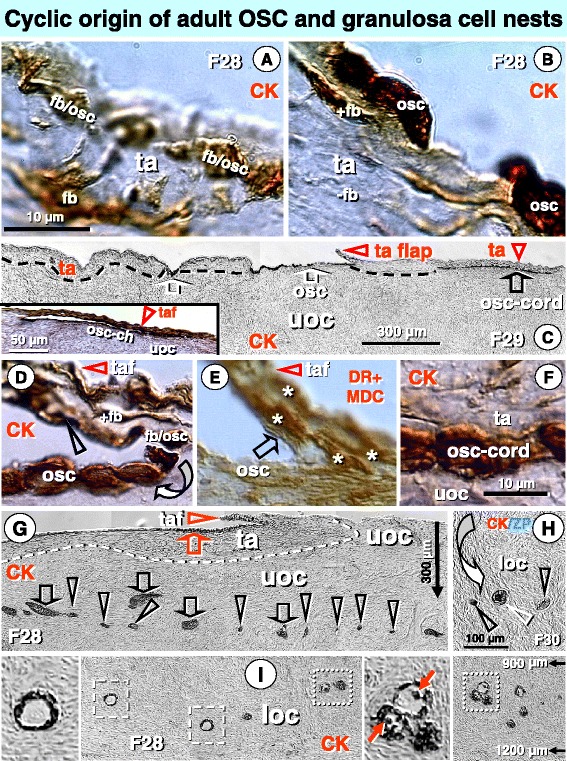
**Cyclic formation of OSC, granulosa cells nests, and presence of new and resting primary follicles during midfollicular phase. A)** Tunica albuginea (ta) fibroblasts (fb) type OSC precursors with CK immunoexpression (brown). Two cells in mesenchymal-OSCs epithelial transition (fb/osc) are apparent. **B)** Appearance of OSCs (osc) is associated with CK depletion (-fb). **C)** Formation of CK+ granulosa cell nests is initiated by a layer of OSC (white arrows) above upper ovarian cortex (uoc). This is overgrown by a developing flap of TA (ta flap or taf in insert) resulting in a bi-layered osc cord (black arrow). Inset shows two layers of the OSC channel. **D)** Detail of OSC flap with CK+ fibroblast type OSC precursors (fb/osc), and OSC development above the upper ovarian cortex (arched arrow). Arrowhead indicates the flap content of OSCs. **E)** A parallel section to (D) showing numerous DR+ MDC (asterisks) in the TA flap. Note DR expression in early OSC (arrow). **F)** Detail of OSC-cord from panel C shows CK+ epithelial cord. **G)** OSC flap (red arrowhead) over a segment of TA (dashed line) covered by OSC layer (red arrow). The OSC cord-derived granulosa cell clusters (black arrows) fragment into granulosa cell nests (black arrowheads). Dashed line indicates a segment of TA covered by OSC epithelium. **H)** Granulosa cell nests (black arrowheads) move by stromal rearrangements (arched arrow) to the lower ovarian cortex (loc) and form new primary follicles (white arrowhead) containing ZP+ oocytes. **I)** Lower ovarian cortex (loc) with new primary (right panel segment) and resting primary follicles (left). Right inset shows the presence of primary Balbiani bodies. Left inset shows lack of Balbiani bodies. Bar in (A), for (A and B), bar in (F) for (D-F). Adapted from Ref. [23]: © Antonin Bukovsky.

Figure 5 demonstrates formation of fresh OSCs differentiating from the TA CK+ fibroblast-like precursors and cyclic emergence of OSC-derived granulosa cell nests for follicular renewal. CK+ fibroblasts in the TA (OSC precursors formed perinatally from fetal OSCs) and their early transformation into adults OSCs under the ovarian surface are presented. When the epithelial type OSCs are formed, the CK staining in their precursors diminishes.

To form granulosa cells, the OSCs at the ovarian surface are overgrown by the TA flap. A completed OSC channel collapses into a bilaminar OSC cord. The OSC formation from CK+ precursors is accompanied by numerous DR+ MDC, and developing OSCs are DR+. Bilaminar OSC cord lying on upper ovarian cortex is covered by the TA.

The process of granulosa cells formation with TA flap and new OSCs at the ovarian surface pretends the fragmentation of granulosa cell clusters into granulosa cell nests, occurring at about 300 microns down from the surface. The granulosa cell nests move by stromal rearrangements to the lower ovarian cortex. At a distance of about 1000 microns from ovarian surface, the follicular renewal is apparent. As expected, the new primary follicles exhibit CK+ primary Balbiani bodies. Resting primary follicles occur at the same distance from ovarian surface, but, unexpectedly, show no Balbiani bodies, possibly due to their complete utilization by the resting primary follicles, which may be required for the further oocyte growth in preantral follicles.

Taking together, the Figure 5 shows four simultaneous processes, which may have a sequential cyclic origin, each of which should be accompanied by a formation of new adult germ cells. The first two of cyclic formations may not succeed to form the new primary follicles. They include development of new OSCs in the first ovarian cycle, formation of granulosa cell nests in the upper ovarian cortex during the second cycle, movement of granulosa cell nests to the lower ovarian cortex and formation of new primary follicles during the third cycle, and development of resting primary follicles, part of which will be destined for the growth of preantral follicles, during the fourth cycle. The development of small antral follicles, suitable for IVM, since they exhibit a secondary Balbiani body formation derived from granulosa cells (see below), can be expected during the fifth cycle, and development of large antral follicles, suitable for IVF, can follow during the sixth cycle. In other words, the effective outcome of *in vivo* treatment after a small blood volume replacement or mononuclear cells transfusion suggested below can be expected after 4-6 ovarian (menstrual) cycles, i.e., 4-6 months, depending on the choosing the IVM or IVF approach.

### Ovarian stem cell cultures

**Figure 6:**
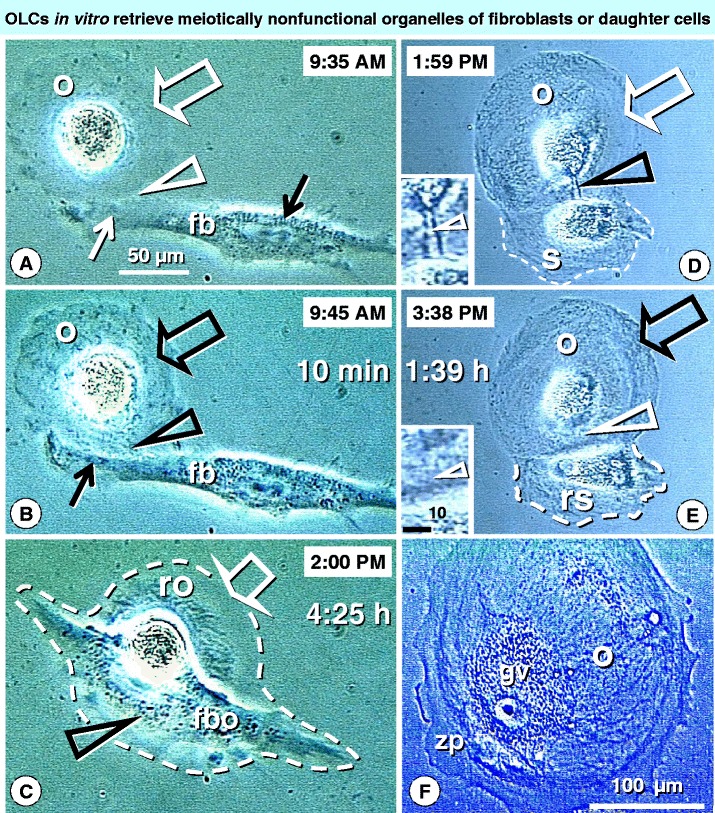
***In vitro*** **developing oocytes are supplied with meiotically nonfunctional organelles from fibroblasts or satellite cells.** Time lapse photography shows that early developing oocytes (o, panel **A**) are low in optically dense cytoplasmic organelles (white open arrow). They can be joined (arrowhead) by fibroblast-like cells (fb), providing additional organelles. Such fibroblast-type cells initially show optically dense organelles close to the nucleus (black solid arrow), but not in the arm extended toward the oocyte (white solid arrow). Within 10 min (panel **B**), however, the optically dense organelles are apparent in the extended arm (solid black arrow) and within adjacent oocyte cytoplasm (black arrowhead) and distant oocyte regions (black open arrow). At 4h 25 min (panel **C**), however, the fibro-oocyte (fbo) hybrid is formed and regressing oocyte (ro) exhibits depletion of organelles (arrow) accumulated by the fibroblast (arrowhead). Alternatively, the developing oocytes (o, panel **D**) deficient in cytoplasmic organelles (white arrow) exploit the satellite cells (s), which are produced by the oocytes themselves. The oocyte is supplied by suicidal satellite cell by a tube like ring canal - (black arrowhead; see inset). In panel **E** the oocyte exhibits enhanced content of the optically dense organelles (black arrow) and the ring canal draining the satellite disappears (white arrowhead - see inset). The satellite cell size is reduced (dashed line) and the perinuclear space is altered (compare with panel **D**). Oocytes enriched by satellites’ organelles (panel **F**) exhibit good morphology [200 micron size, germinal vesicle (gv), and thick zona pellucida (zp)], but are unable to resume meiosis II due to the lack of meiotically functional organelles provided by secondary Balbiani body derived from granulosa cells *in vivo*. Bar in A for A-E. Panel C reprinted from Ref. [26]: © Antonin Bukovsky. Other panels adapted from Ref. [34], with a permission: © Wiley-Liss, Inc.

Oocyte like cells developed in OSC cultures are very immature, since they do not exhibit ZP3 expression and CK positive Balbiani bodies. Observations from time lapse photography [34] (Figure 6) have shown that early developing oocytes have few optically dense cytoplasmic organelles. They appear to be smart and join fibroblast-type cells which provide additional organelles. Such fibroblast-type cells initially show optically dense organelles close to the nucleus, but not in the arm extended toward the oocyte. Within 10 min from the previous photograph, however, optically dense organelles are evidenced in the extended fibroblast arm, within adjacent oocyte cytoplasm, and also in distant oocyte regions. After 4 h and 25 min, however, the fibro-oocyte hybrid is formed and the dominant fibroblast cell collects organelles from regressing oocyte.

The smarter OLCs produce a satellite daughter cell and are supplied of additional organelles with an extended tube. When supply is completed after 1 h and 39 min, the tube disappears and the satellite is regressing. Although such OLCs behavior results in large OLCs with a germinal vesicle (GV), thick ZP, and flat OLCs of 200 microns in diameter, the lack of secondary Balbiani body with granulosa cell organelles enabling the oocyte to respond gonadotropins may be a cause of a failure for meiosis II resumption.

### Perspectives of advanced woman age, premature ovarian failure, or other ovarian infertility etiologies by *in vitro* developed oocytes in ovarian stem cell cultures

The advantage of *in vitro* OLCs development from cultured OSCs is that it is age-independent and does not require the presence of any germ and granulosa cells or ovarian follicles in the ovaries.

Ovarian stem cell cultures do not consist of OSCs alone. They contain also other cell types accompanying OSCs and adjacent TA, since the cellular content is scrapped from the ovarian surface as indicated previously [10]. The following cell types detected immunohistochemically [10,23-25] may, or may not be present: OSC-CK+ fibroblast-like precursors, OSCs, germ cells from asymmetric division of OSCs, germ cells in chromosomal crossover, germ cells in meiosis I division, post-meiosis I migrating germ cells, fibroblasts, Thy-1+ vascular pericytes, CD14 and DR+ MDCs, CD8 and DR+ T cells, endothelial cells, and possibly other cell types.

Unfortunately, the former clinical trial in 2006, accompanied by IVM, was unsuccessful due to the absence of properly differentiated OLCs which attract spermatozoa to be fertilized - reported in Ref. [26], although a single preblastocyst like structure was found after *in vitro* insemination [44]. Nevertheless, *in vitro* cultured OSCs are capable of forming parthenogenetic embryos [26], which may be a source of autologous stem cells, and also autologous neural and neuronal cells after sex-steroid treatment [27].

### Nuclear transfer

To prevent mitochondrial disorders, it has been demonstrated in non-human primates that the mitochondrial genome can be efficiently replaced by spindle-chromosomal complex transfer from one oocyte to an enucleated, mitochondrial-replete egg. The reconstructed oocytes with mitochondrial replacement were capable of supporting normal fertilization, embryo development, and produced healthy offspring [45]. More recently, this group studied the feasibility of mtDNA replacement in human oocytes by spindle transfer (ST). Fertilization rate in ST oocytes (73%) was similar to controls (75%). However, a significant portion of ST zygotes (52%) displayed abnormal fertilization as determined by irregular number of pronuclei. Among normally fertilized ST zygotes, blastocyst development (62%) and ESC isolation (38%) rates were comparable to controls [46]. Recent commentary of John Zhang [47] recommended a revision of GV transfer for the treatment of infertile women with diminished ovarian reserve.

Similar experiments to prevent the transmission of human mitochondrial mutations by mitochondrial gene replacement were performed by another group [48]. The authors reported that defective mitochondrial DNA accidentally transferred with the nuclear genome was initially detected at levels below 1%, decreasing in blastocysts and stem-cell lines to undetectable levels, and remaining undetectable after passaging for more than one year, clonal expansion, differentiation into neurons, cardiomyocytes or ß-cells, and after cellular reprogramming. Stem cells and differentiated cells had mitochondrial respiratory chain enzyme activities and oxygen consumption rates indistinguishable from controls. These results demonstrated the potential of nuclear genome transfer to prevent the transmission of mitochondrial disorders in humans.

### Novel *in vitro* proposals for ovarian infertility treatment

#### Preliminary strategies for *in vitro* approaches

Ovarian stem cells carry imprint from primordial germ cells to develop into distinct cell types including human OLCs [10] or neural stem cells [27]. Such a differentiations just depend on culture medium conditions, and sex steroid components in particular. Development of OLCs was also observed in porcine skin stem cells [29]. The stem cells used for generation of OLCs may have an imprint from primordial germ cells to behave like oocytes, e.g., to increase the content of cytoplasmic organelles by fusion with other cell types present, or from their own daughter cells (see Figure 6). However, they may not exhibit meiosis-I chromosomal crossover mediated by OSC committed CD8/DR+ T cells, meiosis I symmetric division (cytokinesis) mediated by OSC-committed CD14 MDC. Due to the lack of granulosa cells the primary and secondary Balbiani bodies, which are necessary for an early oocyte development and later meiosis II resumption, are absent.

#### Formation of germ cells

For the testing of *in vitro* approaches, OSCs are collected from oophorectomy samples, regardless of the patient’s age [10]. To avoid alteration of OSC cultures by fibroblasts (see Figure 6 and below), the OSC alone should be collected from ovarian surface by sterile cotton swab moistened in culture medium or using blunt edge of a sterile scalpel. Culture processing, tissue culture media, hormonal conditions, and other variables for primary OSC cultures are available in Ref. [10].

Two to four OSC subcultures should be established. To prepare *in vitro* approaches described below for the early stage of OLC proper development, half of the established OSC subcultures may be supported by a small amount of mononuclear cells (including CD14 MDC and CD8 T cells), collected by their separation of mononuclear cells from about 10–20 ml of blood of a fertile ABO blood typing compatible woman, perhaps optimally during the beginning of the midfollicular phase (one to four days after menstruation). An alternative would be to separate mononuclear cells from zero blood typing transfusion bag collected at the same follicular phase period.

If a donor zero blood group transfusion bag is utilized, the remaining collected mononuclear cells can be processed for freezing and stored for utilization in additional OSC cultures.

Donor mononuclear cells can ensure early stages of emerging germ cell maturation, i.e., asymmetric division of OSCs, chromosome crossover, and meiosis I symmetric division - see Figure 1A-C and Ref. [24,26].

#### Formation of granulosa cells

In addition, granulosa cells also originate from OSCs by the influence of DR+ MDC during the same follicular phase period [26]. Interaction of OLCs with autologous granulosa cells may ensure primary Balbiani body formation. For suitability of the culture for IVM, however, the secondary Balbiani body should be formed, like in the small antral follicles (see below). Therefore, self granulosa cells either associate with evolving OLCs, forming primary follicle structures, or additional granulosa cells should be added when some oocytes reach the 80-100 micrometers size and begin to form daughter cells to enrich their cytoplasmic organelles for further growth and maturation.

#### Separation of donor mononuclear cells

The procedure of separating mononuclear cells [49] would exclude the presence of lighter circulating donor germ cells. Their presence in the blood from the early midfollicular phase is, however, unlikely.

#### Collection of ovarian stem cells for a clinical approach

For clinical approaches, OSCs can be collected from infertile women by laparoscopy, as previously reported from the first clinical trial [26].

Use of ultrasound-guided and vacuum-assisted thicker needle may be a simpler alternative for collecting loose OSC from ovarian surface without patient’s bodily anesthesia. This, however, may provide virtually pure OSC, without other cellular components present in TA, which also may be important. That may be fixed with OSC culture support by a small amount of donor mononuclear cells (see above).

### Oocyte-like cell nuclear transfer to donor oocyte

The aforementioned nuclear transfer studies [45-48] utilize enucleated donor metaphase-II (MII) oocytes with normal mitochondrial composition. OLCs develop from OSCs without any interaction with granulosa cells. This procedure is associated with the absence of cytoplasmic organelles required for later maturation. In contrast, transfer of a nucleus (GV) from an OLC to an enucleated donor GV oocyte, which is ready for IVM and still contains cumulus granulosa cells, may represent a reconstructed oocyte capable of IVM and IVF.

### Donor oocyte cytoplasmic transfer to oocyte-like cell

Heide Schatten and colleagues [8] in their contribution to the IVF series dealt with a vital role of mitochondria in oocyte functions. These authors suggested that MII oocytes from women affected by metabolic disorders may be improved by transfer of 10-15% mitochondria from cells exhibiting mitochondrial integrity into mitochondria-impaired oocytes.

As indicated above, the issue that may be solved by transferring a nucleus from an OLC to an enucleated donor GV oocyte which contains cumulus granulosa cells is to provide proper novel cytoplasmic organelles which otherwise would be lacking due to the absence of granulosa cell-derived secondary Balbiani body. The transfer of mitochondria and other organelles from donor MII oocytes to OLCs may be an alternative to the nuclear transfer suggested above.

### Transfer of granulosa cells or their components to an established ovarian stem cell culture

Established OLC cultures usually contain OLCs of various sizes which fail to express ZP proteins, contrasting the ZP expression in oocytes developed in the presence of granulosa cells in ovarian follicles. As shown in Figure 6, larger OLCs easily incorporate additional organelles for continuation of their growth. Since they are unable to interact with granulosa cells, they may either associate with fibroblasts that hybridize and kill the OLCs, or divide to produce daughter cells that support OLC growth although not functional maturation.

Preferably, at the beginning several primary OLC cultures (2 to 4) may be established from scrapped OSCs. In order to induce the development of functional oocytes from OLCs, addition of donor or non-donor (where non-donor means allogeneic source free of charge) granulosa cells, including collection of granulosa cells after oophorectomy (except atretic follicles) in women ≤35 years of age, would be recommended. Granulosa cells from small antral follicles might be preferable. To test the retrieved granulosa cells, they should be added to a small part of the established primary cultures to ascertain the formation of CK positive Balbiani bodies within OLCs. If secondary Balbiani bodies were detected by IHC in large OLCs, some other live subculture may be subjected to IVM and IVF to develop embryos.

### Transfer of granulosa cells or their components to fresh secondary ovarian stem cell cultures during early steps of oocyte reconstruction

**Figure 7:**
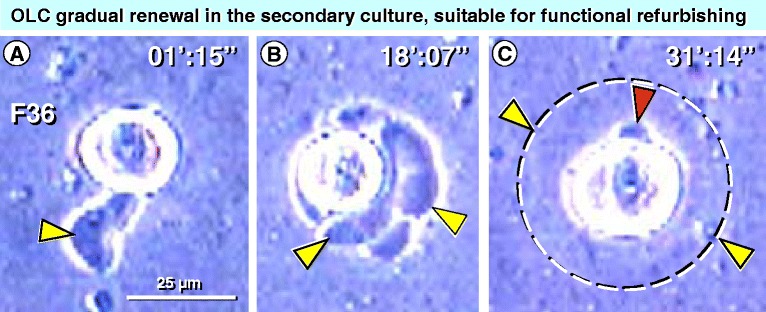
**Time lapse video of oocyte reconstruction in secondary OSC culture. A)** Early developing cell with a cytoplasmic tail (arrowhead). **B)** Multiple cytoplasmic eruptions (arrowheads). **C)** Development of the 50 micron oocyte-like cell (yellow arrowheads indicate cell surface, red arrowhead a polar body). Time in min':sec". Movie segments are available in the Additional file 1: Video S1. Reprinted from Ref. [26]: © Antonin Bukovsky.

Primary OSC cultures (see previous section for the establishment of multiple primary cultures) can be trypsinized and stored frozen for later utilization. Figure 7 shows images from time lapse cinematography of oocyte development in a secondary OSC culture, beginning 4 h after seeding. Video excerpts are available as Additional file 1: Video S1.

The behavior of OLCs in secondary culture shows a new formation of the ooplasm with full volume from the available culture medium and its components. It resembles a situation of oocyte expansion between its capture by granulosa cell nests (see Figure 4A) and early formation of new primary follicle when primary Balbiani body is formed (see Figure 4B). It is possible that if the fresh intact live granulosa cells are added shortly after the secondary culture is seeded, they may contribute to the Balbiani body formation in the reconstructing OLCs during secondary culture. Optimally, a few OLCs and few granulosa cells in a small culture medium volume may be incubated together in a single well using, for instance, 96 well plates.

If unsuccessful to form the Balbiani body after adding granulosa cells, the mixed OLC and granulosa cell culture may be subjected to trypsinization, and after freezing for several days, left to develop as a new mixed secondary culture.

The use of trypsinized granulosa cells without any former culturing, or cocultured with OLCs freshly developed in secondary culture may be another possibility. However, it should be noted that the longer lasting granulosa cell cultures exhibit a dense fibroepithelial cell character [27], possibly due to their reversal to fibroblast type OSC precursors in the TA (see Figure 5A and B). Such cells will not represent functional granulosa cells suitable for the Balbiani body formation.

Finally, a crude (unfiltered) homogenate of uncultured granulosa cells, either fresh or frozen, could be added to the fresh secondary OLC culture. This will enable renewing OLCs to include granulosa cell components from the granulosa cell cytoplasm-enriched culture medium.

Optimally, granulosa cells should be autologous, but it would be unrealistic to obtain such cells in ovarian infertility cases. Other available sources will be cumulus cells from donors. The non-donor source may be cumulus cells from IVM cases. In addition, granulosa cells retrieved after ovariectomy of fertile women ≤35 years of age may be another option (see above).

**Figure 8:**
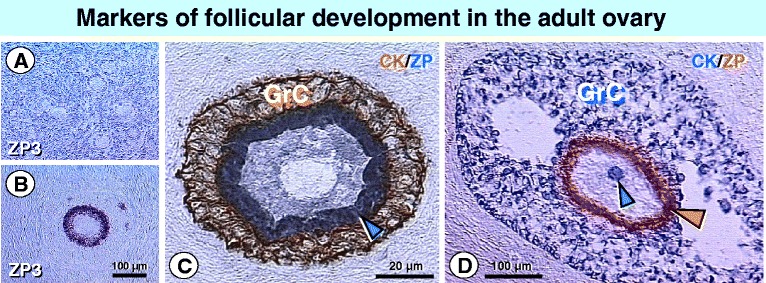
**ZP and CK expression in ovarian follicles.** **A)** Oocytes in resting primary follicles lack ZP3 expression, but express ZP1, ZP2, and ZP4 [41]. **B)** ZP3 is expressed in the oocyte of a growing preantral follicle. **C)** Double color IHC for CK (brown) and ZP (blue) expression in a growing preantral follicle. CK is expressed in granulosa cells (GrC) but no secondary Balbiani body is present in the oocyte. Oocyte surface expresses ZP (blue arrowhead). **D)** Double color IHC for CK (blue) and ZP (brown) expression in a small antral follicle. CK is expressed in granulosa cells (GrC) and in the oocyte secondary Balbiani body (blue arrowhead). Oocyte surface expresses ZP (orange arrowhead).

One of the OLC subcultures should be tested by IHC for the presence of CK positive Balbiani bodies in developing oocytes. In addition, expression of ZP3 at the oocyte surface, which is absent during early follicular development (Figure 8A vs. B) [41], may be included.

As indicated above, primary Balbiani body is formed during follicular renewal, but disappears in resting primary follicles (see Figure 5I). The Balbiani body is also absent in the growing preantral follicles (Figure 8C) but a secondary Balbiani body is formed in small antral follicles (Figure 8D). This is a stage when oocytes initiate the expansive growth and become sensitive to gonadotropins, since they are capable of meiosis II resumption and may be used for IVM.

In culture, such larger OLCs need additional cytoplasmic organelles and they attempt to receive them from the cells present (e.g., fibroblasts) or from own daughter cells (see Figure 6D and E). Such additional organelles are sufficient for advanced OLC growth [26], but they are inappropriate for meiosis II resumption.

The presence of larger OLCs with a poor content of optically dense cytoplasmic organelles (see open white arrow, Figure 6A) may indicate that they originated from germ cells already passing the chromosomal crossover, meiosis I, and preantral follicular development stages. Adding granulosa cells to such OLC culture could be optimal for enabling OLCs to form secondary Balbiani bodies. This may be followed by resumption of meiosis II by IVM and successful IVF.

### Fibroblasts in OSC cultures steal ZP3 expression from OLCs

**Figure 9:**
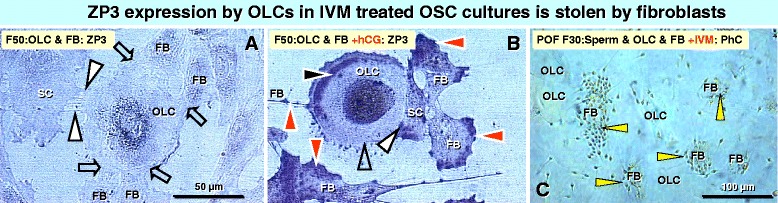
**ZP3 expression by OLCs in IVM treated OSC cultures is stolen by fibroblasts.** **A)** OLCs in untreated OSC cultures show week nuclear ZP3 expression, which is absent in accompanying satellite cells (SC) and fibroblasts (FB). Arrowheads indicate tube like ring canals between OLC and SC. Arrows indicate bindings of FBs to the OLC. **B)** After hCG treatment the OLC exhibits strong nuclear and surface (black arrowhead) ZP3 expression, which is also present in accompanying SC (white arrowhead). The ZP3 expression is stolen by FBs (red arrowheads), leaving the surface of OLC ZP3 depleted (open arrowhead). **C)** The OSC culture from a 30 years old POF female was IVM (FSH+hCG) pretreated and fertilized with the husband's sperm. The phase contrast (PhC) image from a live culture shows that virtually all sperm are associated with fibroblasts instead with OLCs.

Zona pellucida glycoprotein 3 (ZP3) has been recently shown to act as a primary sperm receptor for sperm-egg binding in humans [50]. Figure 9 shows a weak nuclear ZP3 expression, indicating a preparation for cytoplasmic ZP3 synthesis, and no ZP3 expression in associated satellite cell and fibroblasts in untreated OSC culture from a 50 year old human female (F50). The treatment of another F50 culture with hCG caused marked nuclear and surface ZP3 expression in OLC and regressing satellite cell. The accompanying fibroblasts, however, steal the OLC and satellite cell organelles, including ZP3 protein. It is also shown that in FSH & hCG treated OSC culture from POF women in the former clinical trial (see Ref. [26]) the sperm curiously associated with fibroblasts, but not with the OLCs. This suggests that in gonadotropin treated OSC cultures the fibroblasts but not OLCs express the ZP3 glycoprotein.

These observations indicate that OLCs in OSC cultures express LH receptor, which is in vivo derived from primary Balbiani body formed by granulosa cells accompanying formation of new primary follicles (see above). The follicular basement membrane, which is absent in OSC cultures, prevents association of ovarian fibroblasts to drain cytoplasmic organelles from the developing oocyte.

The hCG causes ZP3 expression by OLCs but fibroblasts present in OSC cultures cause functional deprivation of developing OLCs, either by direct association with OLCs , or by association with OLC-satellite cell complexes. Because of that, the presence of fibroblasts in OSC cultures should be avoided.

### What may be next in ovarian tissue cultures?

Laboratory experiments and IVM testing should determine which component(s) of granulosa cells, e.g. luteinizing hormone receptor (LHR) expression in cumulus cells [51], is/are essential for the transition of the immature GV to the MII stage, first polar body formation, and fertilization. To express LHR, the granulosa cells should be pre-treated with follicle stimulating hormone [52]. The presence of essential component(s) in the medium of primary or secondary OLC cultures may enhance maturation of the OLCs. However, such treatment may not ensure the need of additional cytoplasmic organelles, optimally provided by granulosa cells. Nevertheless, the germ and granulosa cells originate from the same OSCs. The presence of essential components in the medium and collection of additional organelles from OLCs daughter cells may be sufficient for the IVM approach.

### Novel *in vivo* proposals for ovarian and testicular infertility treatment

In 2005 Conboy and colleagues studied shared circulatory system between old and young mice (heterochronic parabiosis) and concluded that age-related decline of progenitor cell activity in aged mice can be improved by young mouse serum [53]. More recently H. L. Katcher analyzed studies attempting to restore young function of tissues in aging animals and concluded that “It appears that the plasma of young animals is sufficient to cause cellular rejuvenation of old stem cell populations” [54]. The circulating TGF-beta superfamily member growth differentiation factor 11 (GDF11) declines in aged mice, and treatment with GDF11 reversed age-related cardiac hypertrophy [55]. Factors found in young blood induce vascular remodeling, culminating in increased neurogenesis and improved olfactory discrimination in aging mice [56]. A possibility of heart or brain rejuvenation in older human individuals by plasma content in blood transfusion from young individuals [55-62] had currently received significant press attention [63-66].

The aging of some tissues is, however, more complex, then the effect of blood plasma soluble contents decline. The circulating mononuclear cells home in tissues, to which they have been committed during early ontogeny, and longevity of such commitment depends on the tissue appearance during early ontogeny [43]. The tissues beginning their function very early (during embryonic period, when the immune system is not yet in more advanced development), like the heart, can function for hundred years in humans. The ovary, which primary follicles first differentiate with the assistance of immune system-related cells at six month of intrauterine life, is fully functional till about 38 ± 2 years of age, when the immune functions begin to decline [16].

Immune system related cells cause asymmetric division and differentiation of epithelial [36] and other tissue types [67], including the ovary [34]. The extent of differentiation of distinct tissues is different. Squamous epithelia differentiate completely, up to the apoptosis of superficial cells with the help of immune system components (monocytes, T cells, dendritic cells, and immunoglobulins [36]), and skin is significancy altered in aging individuals due to the diminished epidermal barrier repair associated with a depletion of naive T cells [68]. However, maintenance and repair of some other tissues (skeletal and hart muscles, brain) in young individuals should be prevented from an involvement of the T cells [17]. For repair these tissues in aging individuals, the transfer of blood plasma from young donors containing GDF11 can be sufficient.

Taking together, the heterochronic parabiosis causing tissue improvement in aged mice [53] is not caused by serum soluble factors only, but also, at least in some instances, by circulating mononuclear cells from young individuals. This applies to mammalian female reproductive tissues, including humans, in particular (reviewed in Ref. [34]).

A question arises whether compatible small blood volume replacement (or mononuclear cell transfer without or with blood plasma) from a young healthy fertile and ethnically relevant woman donor, perhaps 300–500 ml, optimally collected during the beginning of the midfollicular phase (one to four days after menstruation), and transfused to infertile women may be sufficient for treating women with ovarian infertility, which still carry ovarian stem cells. If whole blood transfusion is chosen, it should be performed as a small blood volume replacement in order to prevent erythrocyte overdose, i.e. immediately after the corresponding blood volume withdrawal.

Accordingly, small blood volume replacement or mononuclear cell transfer from a young healthy fertile man donor may be useful for treating human testicular infertility.

### Systemic treatment of ovarian infertility by a small blood volume replacement or separated mononuclear cells from young healthy fertile donor women

Circulating immune system related mononuclear cells (MDC and T cells) regulate function of virtually all tissues in the body (reviewed in Ref. [17,36]).

Transfer of mononuclear cells by a small blood volume replacement form young fertile women may cause temporary rejuvenation of the immune, ovarian, and endocrine systems. The plasma content of immune components in the blood, e.g., antibodies, may eliminate eventual persisting follicles that enclose aged oocytes unsuitable for IVF. In contrast, mononuclear cell content may cause follicular renewal consisting of fresh autologous oocytes and granulosa cells.

Although certain limitation for such approach may be the presence of circulating donor germ cells after blood transfer, such cells are not expected to be present during the beginning of midfollicular phase, and even if present, they would not find available uncommitted granulosa cell nests for their transformation into oocytes in primary follicles, and would quickly degenerate (see Figure 4D and E).

Eventually, the donor lighter circulating germ cells may be excluded by the vascular transfer of smaller and heavier mononuclear white blood cells separated from the whole blood. Transfer of mononuclear cells alone, however, would also exclude plasma components present in the blood, including hormonal conditions and plasma proteins during the early midfollicular phase, which also may be important.

To deal with this, transfusion of separated mononuclear cells and blood plasma will be most desirable, since this will include soluble blood proteins and hormone components and exclude unnecessary erythrocytes. In particular, the inclusion of plasma components might be desirable in POF and polycystic ovary syndrome infertile women, and in women lacking the menstrual cycle. Transfer of mononuclear cells (without or with plasma) will not require, due to the lack of erythrocytes, a withdrawal of the of patient’s small blood volume.

Consequently, there are three options to consider:

A) Simple small blood volume replacement
B) Transfusion of separated mononuclear cells
C) Transfusion of separated mononuclear cells and blood plasma

- In cycling infertile women, blood or mononuclear cell transfusion should be optimally performed during the early midfollicular phase. The first new primary ovarian follicles, formed by new oocytes and granulosa cell nests, may occur after a period of two to three months (see Figure 5 comments above). Their growth into antral follicles suitable for ovarian stimulation prior to IVF may require about two to three additional months.

Differentiation of preantral and small antral follicles would require a lasting influence of the spectrum of hormonal and growth factors effects [69], which may become temporarily available in the rejuvenated ovary and endocrine system after mononuclear cell transfer. Optimally, the treatment will cause elimination of old aged follicles with dysfunctional oocytes, resulting in a temporary disappearance of the menstrual cycle before the fresh new follicles reach functional maturity. Follicular renewal may last for several months after the rejuvenating treatment. The new follicle cohorts (**restored ovarian reserve**) and restored ovarian function may last for several years. The experience will show.

The differentiation of some new follicles within ovaries, accompanied by hormonal stimulation for their differentiation into at least small antral follicles may also be considered. This would be sufficient for utilization of oocytes in IVM and fertilization procedures giving pregnancy rates between 22% to 55.6% [70]. Sequential differentiation of the remaining follicles in the new cohort(s) may also be expected.

If needed, additional small blood volume replacement or separated mononuclear-cell transfusion from a different young healthy fertile woman donor may be considered after a 6 month period.

### Systemic treatment of testicular infertility by small blood volume replacement from young healthy fertile donor men

It is important to note again that even with suitable fresh oocytes developed in older women, the sperm of some older men may cause fetal genetic abnormalities [21]. In addition, infertility of human males affects almost a half of infertility cases worldwide [71]. It has been shown that depletion of MDC in one testis of adult rats selectively abolished differentiation of Leydig cells from mesenchymal precursors [72]. Recent findings indicate that CD14 MDC in circulation are involved in a variety of physiological functions other than innate and acquired immune responses, such as repair and regeneration of tissues [73]. It might be possible that small compatible blood volume replacement from young fertile and currently sexually active men may improve the testicular fertility and sperm quality in older or infertile men, and in azoospermia. The same may apply for problematic intracytoplasmic sperm injection cases. This may apply to older couples, where women are treated for ovarian infertility, in particular. The expected temporary testicular rejuvenation should be initiated three months prior conception or fertilization, when the new sperm can be expected to be available [74]. After initiation of the treatment, occasional masturbation is recommended to deplete the old sperm.

### Systemic treatment of other functional diseases by tissue rejuvenation

#### Utilization of small blood volume replacement from young healthy individuals

As indicated above, there is a recent boom dealing with the blood plasma involvement in rejuvenation of aging tissues [53-66]. Here we propose for the first time that blood mononuclear cells are essential for rejuvenation of those tissues, where immune system components participate in an appropriate division and differentiation of tissue stem cells.

The body rejuvenation by functional mononuclear cells and blood plasma from young healthy individuals of the same sex and ethnicity could represent a practical clinical approach causing a decline of *in vitro* methodology in favor of *in vivo* treatment. If needed, functional mononuclear cells from distinct young healthy individuals could be utilized in six month intervals.

For *in vivo* approach it will be important to use small volume blood replacement instead of simple blood transfusion. The blood replacement can be omitted when mononuclear cells, optimally along with the plasma, are used.

Alternatively, such mononuclear cells should accompany *in vitro* methodology and its *in vivo* application. Systemic use of young mononuclear cells from young donor should be applied shortly before the *in vitro* methodology is applied *in vivo*.

### Systemic and local use of honey bee propolis

Improvement of homeostatic functions of the immune system related cells can also be induced by honey bee propolis in animal and *in vitro* models of functional diseases [75-77]. Animal and cell culture experiments indicate that the propolis utilization strengthens the body’s immune system and activates the thymus [78-81]. The immune system itself, including its homeostatic functions [17,67], is significantly deteriorating with the age advancement. Thymic involution associated with age advancement is delayed in human females compared to males [82]. This might be why males are earlier affected by aging compared to females, and why the women live longer due to the better preservation of a role of the immune system in body homeostasis.

Improvement of hypertension and type 2 diabetes, allowing 50% decrease of medications, was observed after oral systemic use of propolis ethanol extract (propolis tincture). Local propolis application totally prevented formation of dental calculus, break off alopecia progression, improved aged skin conditions, and caused regression of varicose veins (unpublished observations). Animal experiments indicated that propolis diet resulted in significant decrease of systolic blood pressure in spontaneously hypertensive rats, but had no effect on normal rat controls [75]. Propolis ethanol extract was reported to attenuate blood glucose and plasma cholesterol in *ob/ob* mice [76]. Ethanolic soluble derivative of propolis extract administered to diabetic mouse models increased the number of immunoregulatory T cells, causing decrease of blood sugar levels [83]. Propolis administered to rats by oral gavage reduced blood glucose levels in diabetes and might be beneficial for the treatment of periodontitis [84]. Propolis flavinoids liposome could effectively activate the cellular and humoral reactivity of immune cells in mice, including proliferation rates of splenic lymphocytes [77].

A question arises, whether propolis can improve ovarian and testicular function. Isolated recent report suggests that toxic effects of methoxychlor on rat ovaries were neutralized by the administration of propolis [85]. Available animal and human studies indicate that propolis have a protective effect and stimulates sperm function [86-91]. The question whether propolis can improve ovarian or testicular age-induced infertility has not been studied yet.

## Conclusions

From the Darwinian point and nature view, it is unacceptable that adult higher vertebrate females are overwhelmingly referred to carry primordial follicles persisting from the fetal period of life, with 15-35 years old “functional” oocytes in human females.

Apparently, human oocytes begin to age after 35-40 years of women’s age, when the number of oocytes and primary follicles begins to significantly decline [12,13]. This is due to the follicular renewal cease, which is caused by the lack of available uncommitted granulosa cell nests [33,34]. Formation of new granulosa cells is immune system dependent (see above). Therefore, a lack of formation of granulosa cells is a consequence of the age-induced alterations of homeostatic immune system functions [17] beginning in humans from the age of 35 years [16]. At the same age, the ovarian infertility begins to sharply increase [7].

The data presented show that like in the plants, invertebrates, lower vertebrate females, and higher vertebrate males, the formation of new germ cells and gametes persists in women during the prime reproductive period in adulthood.

In adult fertile human females, the new germ cells are derived by asymmetric division from CK+ OSCs, exhibit depletion of CK and MHC-I expression, show meiosis I events, and contribute to the cyclic follicular renewal. Bipotential CK+ OSCs are also a source of new granulosa cells required for the follicular renewal. The follicular renewal is also dependent on support of circulating blood mononuclear cells. They induce intermediary stages of meiosis (metaphase I chromosomal duplication and crossover, telophase, and cytokinesis) in emerging ovarian germ cells, as for the first time demonstrated here. They also induce formation of granulosa cells, and stimulate follicular growth and development. These functions of CK+ OSCs and blood mononuclear cells are already present in midpregnancy fetal ovaries. The only role of primordial germ cells is to imprint the CK+ OSCs (and stem cells in some other tissues - e.g. skin) toward a potential differentiation into fetal and adult germ cells. Similarly to embryonic stem cells, the OSCs are also capable to differentiate into other cell types, depending on the local *in vivo* or *in vitro* conditions.

Based on these measures, the current nomenclature should be revised as follows: **Fetal** and **Adult ovarian germ cells**, **New primary follicles**, and **Resting primary follicles**.

The high demand by older women to conceive and give birth to their own children together with the increased infertility after the age of 40 years raises a problem to be solved by IVF/IVM approaches. The same applies for younger women with POF, or other younger women with ovarian infertility.

*In vitro* development of OSC-derived OLCs is independent of the woman’s reproductive aging. The clinical utilization of OLCs in POF women is, however, still unsuccessful. Among the proposed strategies to solve this matter and enable pregnancy in women with ovarian infertility, nuclear transfer to a donor oocyte or cytoplasmic transfer from a donor oocyte raise ethical and social concerns in some countries in terms of three parent pregnancy [92], but it can be used in other countries. In addition, these techniques should be worldwide tolerable in women considering to become pregnant from oocyte/embryo donation, which is an accepted event of the three parent pregnancy.

Utilization of intact granulosa cells and/or their purified components in OLC cultures may be essential. A better understanding of granulosa cell component(s) is needed (e.g. LHR) for oocyte maturation from the meiosis I to meiosis II stage. Passage it/them to the medium of the fresh secondary OLC culture in a purified form, may be followed by successful IVM and IVF. Additional observations presented for the first time here also indicate that presence of fibroblasts in OSC cultures should be avoided, since they steal cytoplasmic organelles, including ZP3 glycoprotein needed for the sperm-egg binding, from the developing and hCG stimulated OLCs.

Ovarian infertility may possibly be treated also without OLC cultures, by compatible small blood volume replacement or transfer of mononuclear cells separated from the blood of a young healthy fertile woman donor, and eventually accompanied by the blood plasma. This may renew the “steps required for follicular renewal in adult human ovaries” and renew the ovarian reserve, which may function for several additional years (see above).

If the formation of new primary follicles and their differentiation in adult human ovaries of women with ovarian infertility took place after compatible small blood volume replacement or transfer of mononuclear cells (without or with blood plasma) from young healthy fertile women, it would represent a practical clinical approach causing a decline of *in vitro* methodology in favor of *in vivo* treatment, as proposed by Deepa Bhartiya and colleagues [9] in the IVF article series [1].

It is proposed that the simple and inexpensive *in vivo* resumption of follicular renewal should be attempted in women with ovarian infertility as the first step - for women considering pregnancy from oocyte/embryo donation, in particular. Although unlikely, it would be important to determine whether donor mononuclear cells can cause a three parent baby. If so, each blood transfusion woman recipient, and possibly also man, should be made aware of that.

Infertile patients attending IVF centers may request a suitable relative or friend with matching ABO blood and Rh factor typing for proper ovarian cycle period blood collection and small blood volume replacement at a transfusion unit. Optimally, a purchase of compatible blood or separated mononuclear cells (without or with blood plasma) collected from young healthy fertile women during early midfollicular phase from transfusion units may be negotiated by IVF centers, and small blood volume replacement or mononuclear cell transfer performed at the similar ovarian cycle period in cycling infertile patients. Interestingly, a successful pregnancy has been reported after allogeneic bone marrow transplantation in a patient with chronic myeloid leukemia [93].

Some of the novel proposed *in vitro* approaches (see above) may be used as a second option, which can provide much faster (within four to six weeks) treatment of ovarian infertility. These approaches may generate oocytes suitable for IVM/IVF outside of the more complex *in vivo* body conditions, such as formation of human granulosa cell nests, their transfer from the ovarian surface to the lower ovarian cortex, vascular transport of germ cells, and formation of new primary follicles by association of developing small oocytes with intravascular granulosa cell nests [23,24,26,34,35]. Some of these processes may *in vivo* be under autonomic innervation control [17,34,94], which may not be possible to restore by mononuclear cell transfer alone.

Previously missing intermediary stages of meiosis in adult mammalian ovaries [95] are now for the first time demonstrated here in humans, evidencing that the germ cell and follicular renewal exists during the prime reproductive period. Follicular renewal is dependent on the support of OSC-committed circulating blood mononuclear cells, which are required for renewal of functional germ and granulosa cells from OSCs. The granulosa cell-committed mononuclear cells can be depleted in older women. They may also be absent in other cases of ovarian infertility, along with, or without the absence of mononuclear cells committed for renewal of adult germ cells.

A combination of blood or separated mononuclear cells transfusion with laparoscopic (or ultrasound-guided and vacuum-assisted) collection of OSCs for *in vitro* cultures several days thereafter, is a third possibility. This may allow a collection of autologous new ovarian germ cells passing the crossover and meiosis I division in the ovarian TA, and freshly developed granulosa cells for *in vitro* processing.

The compatible small blood volume replacement from young fertile and currently sexually active men may also be a suitable and easy approach for the treatment of testicular infertility and sperm quality improvement in older or other affected men, in azoospermia, and for problematic intracytoplasmic sperm injection cases. This will apply to older couples, where the women are treated for ovarian infertility, in particular. The testicular rejuvenation should be initiated three months prior conception or *in vivo/in vitro* fertilization.

The body rejuvenation by a compatible small blood volume replacement from young healthy individuals of the same sex and ethnicity could represent a practical clinical approach for human functional diseases, causing a decline of *in vitro* methodology in favor of *in vivo* treatment. If needed, compatible small blood volume replacement or mononuclear cell transfer from distinct young healthy individuals could be utilized in six month intervals for repair of young altered or aged reproductive and other tissue functions. Alternatively, such mononuclear cells could accompany *in vitro* methodology and its *in vivo* application.

Systemic and local use of honey bee propolis, strengthening the homeostatic immune function in aging individuals, is another option for rejuvenation of tissues in older individuals. The role of propolis in the treatment of age-associated ovarian and testicular infertility has not been, however, comprehensively studied yet.

## Additional file

Additional file 1: Video S1. Time lapse oocyte development (one of the total 31 min) launched 4 h after seeding the secondary OSC culture.

## Abbreviations

DR: Major histocompatibility complex class II
ESC: Embryonic stem cells
GDF11: Growth differentiation factor 11
GV: Germinal vesicle
IHC: Immunohistochemistry
IVF: *In vitro* fertilization
IVM: *In vitro* maturation
LHR: Luteinizing hormone receptor
MDC: Monocyte-derived cells
MII: Metaphase-II
MHC: Major histocompatibility complex
OLCs: Oocyte like cells
OSE: Ovarian surface epithelium
OSCs: Ovarian stem cells
POF: Premature ovarian failure
ST: Spindle transfer
TA: Tunica albuginea
ZP: Zona pellucida
